# Recent Trends in Marine Phycotoxins from Australian Coastal Waters

**DOI:** 10.3390/md15020033

**Published:** 2017-02-09

**Authors:** Penelope Ajani, D. Tim Harwood, Shauna A. Murray

**Affiliations:** 1 Climate Change Cluster (C3), University of Technology Sydney, Sydney, NSW 2007, Australia; shauna.murray@uts.edu.au; 2 Cawthron Institute, The Wood, Nelson 7010, New Zealand; tim.harwood@cawthron.org.nz

**Keywords:** harmful algae, ciguatoxins, paralytic shellfish toxins, okadaic acid, domoic acid, palytoxins, karlotoxins, tetrodotoxin, maitotoxin, palytoxin

## Abstract

Phycotoxins, which are produced by harmful microalgae and bioaccumulate in the marine food web, are of growing concern for Australia. These harmful algae pose a threat to ecosystem and human health, as well as constraining the progress of aquaculture, one of the fastest growing food sectors in the world. With better monitoring, advanced analytical skills and an increase in microalgal expertise, many phycotoxins have been identified in Australian coastal waters in recent years. The most concerning of these toxins are ciguatoxin, paralytic shellfish toxins, okadaic acid and domoic acid, with palytoxin and karlotoxin increasing in significance. The potential for tetrodotoxin, maitotoxin and palytoxin to contaminate seafood is also of concern, warranting future investigation. The largest and most significant toxic bloom in Tasmania in 2012 resulted in an estimated total economic loss of ~AUD$23M, indicating that there is an imperative to improve toxin and organism detection methods, clarify the toxin profiles of species of phytoplankton and carry out both intra- and inter-species toxicity comparisons. Future work also includes the application of rapid, real-time molecular assays for the detection of harmful species and toxin genes. This information, in conjunction with a better understanding of the life histories and ecology of harmful bloom species, may lead to more appropriate management of environmental, health and economic resources.

## 1. Introduction

Many people suffer food insecurity throughout the world, and aquaculture is the fastest growing food production sector across the globe [1]. Harmful algal blooms (HABs) and their associated phycotoxins, however, have important economic, health and environmental effects. Global fisheries production is valued at US$91.2 billion, with 110 million tonnes of fish provided for human consumption, 47% of which is derived from aquaculture [2]. Each year, Australia harvests around 227,000 tons of seafood, representing a harvest value of $2.2 billion. The commercial and health risks posed by marine biotoxins to this industry have been specifically recognized by the sector [3] with one single event in Tasmania, Australia, in 2012 estimated to have cost AU$23 million [4].

Marine biotoxins are chemical compounds produced via secondary metabolic pathways by certain microalgae, notably dinoflagellates and diatoms. These contaminants can bioaccumulate in fish, crabs, lobster, abalone or filter-feeding bivalves (shellfish), such as mussels, oysters, scallops and clams, and cause poisoning to seafood consumers. Approximately 60,000 human intoxications occur per year worldwide, with an overall mortality of ~1.5% [5]. There are seven major poisoning syndromes caused by phycotoxins: paralytic shellfish poisoning (PSP); diarrhetic shellfish poisoning (DSP); neurotoxic shellfish poisoning (NSP); amnesic shellfish poisoning (ASP); azaspiracid shellfish poisoning (AZP); ciguatera fish poisoning (CFP); and clupeotoxin fish poisoning (CLP). For each of these conditions, the clinical symptoms include, but are not limited to, gastrointestinal (nausea, vomiting, diarrhoea) and/or neurological (tingling, headaches, dizziness, hallucinations, seizures, death) consequences.

Harmful marine microalgae and their potential risks to food safety and/or market access of commercially-produced seafood are a growing concern in Australia. The types of poisoning syndromes already encountered can be divided into those that have impacted humans or the marine food chain through the ingestion of seafood, those that have caused the deaths of marine life or those causing human skin irritations or breathing difficulties [6]. With the regular monitoring of waterways, aquaculture and wild fisheries, there has been a suite of phycotoxins emerging over the past decade. The aim of this review is to present and discuss these emerging phycotoxins, their causative microalgal species and, if known, their distribution and seasonal occurrence. Included are saxitoxin, okadaic acid, palytoxin, ciguatoxins, maitotoxins and each of their derivatives. We also discuss the potential that tetrodotoxin may be a phycotoxin, the fish killing toxins karlotoxins and the recently identified potential for fish killing activity due to toxins from *Amphidinium* spp., in particular luteophanols, amphidinols and amphidinolides. Furthermore, domoic acid, the only toxin to be produced by diatoms around the world, continues to be a concern as a seafood contaminant in Australia [7].

## 2. Paralytic Shellfish Toxins

Paralytic shellfish toxins (PSTs) are phycotoxins produced by marine dinoflagellates (phytoplankton) that cause paralytic shellfish poisoning, a potentially fatal human illness. Early symptoms of PSP can include tingling of the lips and tongue and progress to fingers and toes, loss of muscle control (including chest and abdomen) and difficulty breathing. With high toxin exposures, death in human seafood consumers has occurred in less than 30 min (http://www.doh.wa.gov, accessed 26 November 2016).

PSTs represent a diverse class of potent neurotoxins produced from dinoflagellates belonging to the genera *Alexandrium*, *Gymnodinium* and *Pyrodinium* that naturally accumulate in filter-feeding shellfish. They are guanidinium derivatives, with saxitoxin being regarded as the parent compound (Table 1). More than 50 related PST analogues have been described to date with varying potencies observed from toxicological investigations [8]. Monitoring for this toxin class traditionally required the mouse bioassay (AOAC 959.08), but in recent times, there has been movement away from animal models for both ethical and technical reasons. Several instrumental methods of analysis have achieved international accreditation and have been implemented for regulatory monitoring purposes, including in Australia. These methods include pre-column oxidation with fluorescence detection (AOAC 2005.06) [9] and post-column oxidation with fluorescence detection (AOAC 2011.02) [10].

For Australian waters, *Alexandrium* is the most challenging genus, both from taxonomic and industry/regulator perspectives. Species of the *Alexandrium tamarense* species complex [11] overlap or are identical in their morphological features, despite the fact that their toxicity can be highly variable and patchy (see [12] for a summary of the toxin profiles of identified strains of the former *Alexandrium tamarense* species complex, including their varying quantities and diverse toxin profiles). Species that produce PSTs in Australia to date are *Alexandrium pacificum*, *Alexandrium australiense*, *Alexandrium fundyense*, *Alexandrium minutum* and *Gymnodinium catenatum*. The main cause of PST episodes in Australia to date has been *Alexandrium pacificum* (as *A. catenella*) [13] and, more recently, *Alexandrium fundyense* (as *A. tamarense*) [14]. In 2012, Japanese import authorities (Ministry of Health, Welfare and Labour) recalled a shipment of blue mussels (*Mytilus galloprovincialis*) derived from the east coast of Tasmania due to the presence of paralytic shellfish toxins at levels many times greater than the maximum permissible level. This bloom event was Australia’s largest and most significant PST event to date, resulting in widespread harvest closures of mussels, oysters, scallops, rock lobster and abalone over a period of six months along 350 km of coastline and total economic losses of ~$23M [4,14]. Bloom isolates were characterized by DNA sequencing based on the LSU-rDNA (large subunit), ITS-rDNA (internal transcribed spacer) and the saxitoxin (STX) synthetase SxtA1/A4 regions and were confirmed as *Alexandrium fundyense* (previously *A. tamarense* Gp 1), a species not previously reported from Australasia. Moreover, all isolates of *Alexandrium fundyense* were found to produce PSTs with the main toxin analogues identified as the *N*-sulfocarbamoyl toxin C1/2 and gonyautoxin (GTX)1/4, low proportions of neosaxitoxin (NEO), *N*-sulfocarbamoyl toxin C3/4 and traces of GTX2/3 and decarbamoyl gonyautoxin (dcGTX)2/3, with an eight-fold variation in STX content (8–65 fmol·cell^−1^) among strains [14]. Three significant toxic genotypes (*A. fundyense*, *A. pacificum* and *P. australiense*) have now been identified from Tasmanian waters, with PST toxin profiles significantly different between the three species [15]. *A. australiense* produces primarily low toxicity GTX5 and GTX6 (ranging from 0.7 to 3.0 pg·cell^−1^ STX eq.); *A. pacificum* yields mostly GTX5, GTX6, GTX1/4 and C1/2 (ranging from 2.3 to 4.0 pg·cell^−1^ STX eq.); while *A. fundyense* reveals the presence of higher toxicity analogues (e.g., GTX1/4 and GTX2/3), as well as C1/2 (ranging from 5.9 to 15.3 pg·cell^−1^ STX eq.) [15]. 

In 2015 and 2016, once again, species of the *A. tamarense* species complex were found in Tasmania in bloom abundances along the east coast, concomitant with high levels of PSTs in shellfish (max concentration of 24 mg·kg^−1^ in mussels collected from Great Oyster Bay., TSQAP unpublished data). Three people were treated, and two were hospitalised (http://www.abc.net.au/news/2015-10-06/two-people-treated-in-tas-hospital-for-shellfish-poisoning/6832220). These were the first reported cases of human illness due to PSP in Australia in 30 years (http://www.abc.net.au/news/2015-10-08/toxic-algae-poisoning-tasmania-could-harm-businesses-scientist/6835066). At present, it is not yet clear which species of the *A. tamarense* species complex was responsible for these toxins, and their toxin profiles are the subject of current research. 

*Alexandrium minutum*, *A. pacificum*, *A. ostenfeldi* and *A. australiense* are known to be distributed along the east coast of mainland Australia from northern NSW to Victoria [12,13,14,16,17], whilst *A. fundyense* has only been identified in Tasmania thus far [14]. The seasonal occurrence for *A. pacificum* is most commonly in spring/summer, *A. fundyense* in winter/spring and *A. minutum* in early summer/autumn. The seasonal occurrence for *A. australiense* in Australian coastal waters is, as yet, unknown [6]. 

With certain lines of evidence suggesting that the toxic dinoflagellate *Gymnodinium catenatum* was introduced into Tasmanian waters relatively recently (after 1972), via ship ballast water discharge from vessels originating most likely from Japan and South Korea (and less likely Europe) [18], this species has regularly and significantly bloomed in the Derwent and Huon Estuaries. In particular, blooms in 1985/1986 led to widespread closures of the local shellfish industry for several months [19]. Since then, *G. catenatum* has caused small localized annual blooms in Tasmania and, more recently, a large PST incident in 2011, which led to PST uptake in mussels and abalone [20]. Another relatively recent discovery is that Australian strains of *G. catenatum* were found to produce a newly-discovered sub-class of paralytic shellfish toxins, the guanylyl cyclase GC toxins, which are hydroxy benzoate derivatives of saxitoxins. These compounds bind strongly to sodium channels and, combined with their lipophilic nature, have the potential to contribute significantly to sample toxicity [21].

Blooms in Tasmanian waters tend to occur during the period from December–June, in water temperatures of 12–18 °C [22]. In New South Wales, *Gymnodinium catenatum* has also been found sporadically at several estuarine sites: Manning River, Brisbane Water, Hawkesbury, Jervis Bay, Tuross Lake, Nelson Lagoon and Merimbula Lake [16]. Generally, it has been present in low abundances, although it did exceed the health department’s action limits four times between 2005 and 2009 in New South Wales [16]. 

## 3. Diarrhetic Shellfish Toxins

Even at very low cell densities (<10^3^ cells·L^−1^), the dinoflagellate genera *Dinophysis* and *Prorocentrum* can produce phycotoxins that cause “diarrhetic shellfish poisoning” (DSP), a type of gastroenteritis in seafood consumers [23,24,25]. Many causative polyether toxins have been identified from these organisms, including okadaic acid (OA), dinophysistoxin-1 (DTX-1) and dinophysistoxin-2 (DTX-2) (Table 1). Many esters of okadaic acid and the dinophysistoxins are formed by conjugation of the terminal carboxylic acid group with poly-hydroxylated, sulphated or unsaturated alcohols. In shellfish, a proportion of the toxins are acylated at the C-7 hydroxyl group with long-chain fatty acids, forming derivatives collectively known as dinophysistoxin-3 (DTX-3). OA and DTXs inhibit serine/threonine protein phosphatases 1 (PP1) and 2A (PP2A) [26,27], increasing protein phosphorylation, which in turn affects several cellular processes, such as metabolism, cytoskeletal maintenance, gene transcription, cell division, membrane transport and secretion [28]. Dinoflagellates of the genus *Dinophysis* also produce neutral polyether lactones known as pectenotoxins (PTXs) (Table 1). More than 20 PTX analogues have been described. Some are synthesized by the microalgae; some are formed through metabolism after uptake by shellfish; while others appear to be artefacts produced during the extraction process. Pectenotoxins have little or no oral toxicity, however, and thus, their threat to public health is still uncertain [29].

Species of *Dinophysis* and *Prorocentrum* are distributed worldwide. Significant DSP episodes have occurred worldwide including in Canada [30], Chile [31], Japan [32], Spain, Portugal and Norway [24], and as such, species of Dinophysis are the focus of many harmful algal monitoring programs throughout the world. *Dinophysis acuminata* is the main agent of DSP events around the world, with some strains producing only PTX, others only OA, others DTX1 and PTX2 or a mixture of OA, DTXs and PTXs (Intergovernmental Oceanographic Commission-UNESCO and the references therein http://www.marinespecies.org/hab/). 

*D. acuminata* has been responsible for three major DSP events in Australia to date, although no fatalities have been recorded. In 1997, *D. acuminata* (and *D. tripos*) was implicated in the contamination of pipis (*Plebidonax deltoides*) in New South Wales [33]. This incident resulted in 102 people being affected and 56 cases of gastroenteritis reported. In March 1998, a second outbreak was reported, in which 20 cases of DSP poisoning were reported [34]. In December 2003, another *D. acuminata* bloom was detected in the Eyre Peninsula, South Australia [35] (Table 2).

During 2003–2004, a high seasonal abundance of both *Dinophysis acuminata* and *D. fortii* (up to 7380 cells·L^−1^ for *D. acuminata*, 500 cells·L^−1^ for *D. fortii*) in Sullivan’s Cove, Tasmania, was associated with the detection of okadaic acid (OA) + dinophysistoxin-1 (DTX-1) in the digestive gland of non-commercial mussels (*Mytilus edulis*) [28]. The diarrhetic shellfish toxins (DSTs) pectenotoxin-2 (PTX-2), PTX-2 seco acids and 7-epi-PTX-2 SA were also detected in the mussels. Further analysis showed toxin profiles of individual species of *D. acuminata* and *D. fortii* to be 10-fold more toxic than the same species from other countries [28]. 

*Dinophysis* species are common in Australian coastal waters, but rarely abundant [58]. Whilst the highest abundance of *D. acuminata* has been observed in spring (max. abundance 4500 cells·L^−1^), *D. caudata*, on the other hand, shows the highest abundance in the summer to autumn (max. 12,000 cells·L^−1^), highlighting the species-specific seasonality of this toxic group [58].

## 4. Palytoxins

In the early 1970s, “palytoxin” (PLTX) was isolated from a *Palythoa* species from Hawaii [59]. Palytoxin has since been isolated from *Palythoa* sp. from Japan and the Caribbean and may be responsible for clupeotoxin fish poisoning (CLP). Other structurally-related compounds have been identified from other *Palythoa* sp. extracts, and these include homopalytoxin, bishomopalytoxin, neopalytoxin, deoxypalytoxin and 42-OH palytoxin [60]. Palytoxin itself is one of the most complex and toxic non-proteinaceous natural products known with a molecular formula of C_129_H_223_N_3_O_54_ and an extremely low LD_50_ (mouse, acute i.p.) of less than 1 mg·kg^−1^ [61,62] (Table 1). 

Palytoxin derivatives are now known to be produced by the epi-benthic or epi-phytic dinoflagellates of the genus *Ostreopsis*, which has a wide global distribution in temperate and tropical waters [63]. The number of known palytoxin-like analogues now approaches 20, including the structurally-related ostreocin-D, ovatoxins a–k and isobaric palytoxin [63,64,65,66,67,68,69]. These compounds are able to transfer through the marine food chain and can result in human illnesses through consumption of contaminated seafood. Evidence of respiratory distress through exposure to aerosolized toxins has also been documented [70]. 

**Figure 1:**
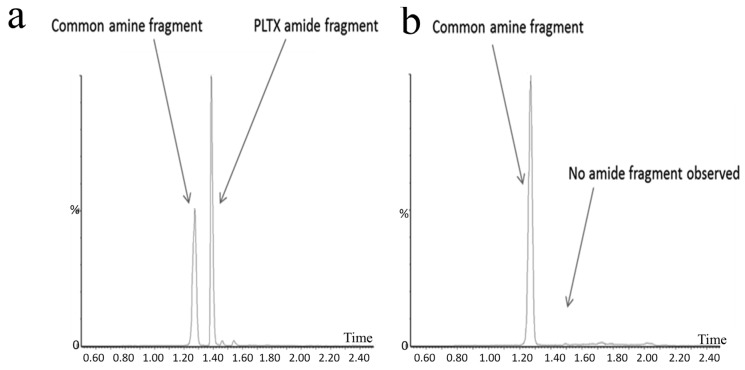
Extracted ion chromatograms from the solid phase extraction and on-column oxidation of (**a**) Palytoxin (PLTX) standard (50 ng·mL^−1^) and (**b**) *Ostreopsis* cf. *siamensis* (Strain identification CAWD203) from Merimbula, Australia [71].

Recently, *Ostreopsis* cf. *siamensis* isolated from Australian coastal waters was examined for the first time for the presence of palytoxin-like compounds using a novel quantitative analytical method [42]. The method uses LC-MS/MS to analyse substructures generated by oxidative cleavage of vicinal diol groups present in the intact toxin. Oxidation of palytoxins, ovatoxins or ostreocins using periodic acid generates two nitrogen-containing aldehyde fragments: an amino aldehyde common to these toxins and an amide aldehyde that may vary depending on the toxin type. An extract generated from the Australian *Ostreopsis* cf. *siamensis* clearly showed the presence of the amino aldehyde fragment. The palytoxin amide fragment was not, however, detected, suggesting that another structurally-related analogue gave rise to the amine fragment observed (Figure 1) [71]. Ongoing investigations are being conducted to determine the structure of this analogue. Furthermore, the total amount of palytoxin-like compounds was determined as 4.62 ng (total of 26,700 cells), resulting in an estimate of 0.17 pg·cell^−1^. Additionally, the LD_50_ of *Ostreopsis* cf. *siamensis* extract by intraperitoneal injection in mice was revealed as 25.1 mg·kg^−1^ (95% confidence interval of 14.0–33.2 mg·kg^−1^) [71].

In another study investigating the hidden diversity of *Ostreopsis* collected from Australia’s Great Barrier Reef, toxin presence (both the amino aldehyde fragment, common to all known PLTX, ovatoxin and ostreocin analogues, as well as the amide aldehyde fragment, thereby confirming the presence of PLTX-like analogues) was only confirmed for *O. ovata* (total amount of PLTX-like analogues estimated as 1.8 pg·cell^−1^), while no PLTX-like analogues were detected from cellular isolates of *O.* cf. *siamensis* or *O. rhodesae* sp. nov. strains. *O. rhodesae* sp. nov., however, was shown to be toxic to fish gill cell lines, warranting further investigation into this newly-described species [43]. The seasonality and potential of these three species to produce harmful blooms in Australian waters remains unknown.

## 5. Ciguatoxins and Maitotoxins

Ciguatera fish poisoning (CFP) is the most common, non-bacterial human illness associated with seafood consumption [72]. The poisoning syndrome is undoubtedly under reported, due to the remoteness of affected individuals and the fact that clinical diagnosis is difficult due to the variable symptoms ranging from neurological, gastrointestinal to cardiovascular [73]. CFP is prevalent in tropical and sub-tropical waters of the South Pacific Ocean, and it affects many of the indigenous populations that inhabit these islands, both populated and remote. CFP is caused by consumption of reef fish containing ciguatoxins (CTX), which are complex fat-soluble toxins produced by micro-algae within the *Gambierdiscus* genus (Table 1). These toxins enter the food chain through herbivorous fish that graze micro-algae and are then bio-accumulated and bio-transformed to more toxic forms as they move up the food chain. *Gambierdiscus* are epiphytic dinoflagellates, well known in tropical reef areas, and although their increasing presence in temperate Australia has now been documented [16,74], a species that consistently produces CTXs has yet to be identified from Australian waters [74,75,76].

*Gambierdiscus* species produce a range of polyether toxins, with ciguatoxins the most extensively studied [77,78,79]. Ciguatoxins isolated from *G. polynesiensis* culture extracts include CTX3C, 49-epi-CTX-3C (synonym: CTX-3B), CTX-4A and CTX-4B. The molecular structures of ciguatoxins found within fish vary with location. To distinguish them, a prefix is typically added to the name: P for compounds from the Pacific (e.g., P-CTX-1B), C for compounds from the Caribbean (e.g., C-CTX-1) and I for those from the Indian Ocean. CTXs are fat-soluble, thermally-stable, cyclic polyether ladder compounds. They are potent neurotoxins that act on sodium channels. As of 2011, 23 ciguatoxin derivatives from the Pacific had been identified, and the structures of the Caribbean ciguatoxin C-CTX-1 and its epimer C-CTX-2 had been determined. Several ciguatoxins have been detected in fish caught in the Indian Ocean, although these have not yet been fully characterized. P-CTX1B (or CTX-1 as described by [80], originally isolated from moray eels) is commonly found in ciguatoxic carnivorous fish from the Pacific [44,47]. The current FDA guideline for CFP is now listed as 0.01 ppb for Pacific ciguatoxin and 0.1 ppb for Caribbean ciguatoxin (USFDA guidelines April 2011, http://www.fda.gov/downloads/Food/GuidanceRegulation/UCM251970.pdf). These levels are extremely low, and in the case of Pacific ciguatoxin, exceed the current capability of many analytical methods and instrumentation.

*Gambierdiscus* species also synthesize other toxins, including gambieric acids [81], gambierol [82], gambierone [83] and maitotoxins [84,85]. Maitotoxin (MTX-1) itself is a water-soluble cyclic polyether ladder marine toxin that was first described in the mid-1970s (Table 1). Its complex molecular structure and stereochemistry were subsequently determined in the 1990s. MTX represents the largest natural non-biopolymer compound known with a molecular formula of C_164_H_256_O_68_S_2_Na_2_ (MW 3425.9 g·mol^−1^) and is an extremely potent calcium channel inhibitor with an LD_50_ of 0.05 μg·kg^−1^ via intraperitoneal injection into mice [86]. Australian researchers have described other maitotoxins (MTX-2, MTX-3) from different strains of *Gambierdiscus* sp. isolated from Queensland, and these display high levels of toxicity in in vitro systems [84]. Although MTX-1 appears to have a low tendency of accumulating in fish flesh, as compared to stomach or intestines [87], its possible role in CFP cannot be disregarded, as eating non-eviscerated fish is a common practice in many Pacific Island nations. The anionic sulphate esters present in the structure of the various maitotoxins make them amenable to detection and quantification by LC-MS. More research is essential to understand the exact role of MTXs in CFP including its mode of action and target in mammalian cells.

Whilst *Gambierdiscus belizeanus*, *G.* (now *Fukuyoa*) *yasumotoi* [76], an unknown *Gambierdiscus* sp. genotype (A213) [88] and *G. carpenteri* [74] have been found in Australian waters, the identification of *Gambierdiscus* species producing CTXs in Australia has been hampered by several uncertainties and difficulties, and no identified strains that consistently produce known CTX analogues have yet been confirmed. Furthermore, whilst these species have been found at a few sites on the east coast of Australia, mainly in Queensland, their distribution and seasonal occurrence is as yet unknown [6]. In a very recent study, however, a new species of *Gambierdiscus* (*G. lapillus* sp. nov.) was isolated from Heron Island. It did not produce maitotoxin (MTX-1) or the known algal-derived ciguatoxin analogues (CTX3B, 3C, CTX4A, 4B), but did produce putative MTX-3 and a number of unknown compounds, which may include undescribed congeners of CTX [75].

Whilst CFP is common in tropical and sub-tropical areas in Australia (65 outbreaks and >283 cases from 2001 to 2010 Australia wide, [89]), despite a likely reporting rate of ~20%, it has only been in recent years that the most southern confirmed sources of CFP in Australia have been reported [90]. Prior to 2014, there had been only one documented outbreak of ciguatera fish poisoning from fish caught in the state of New South Wales (in 2002), and yet since 2014, outbreaks of CFP have been reported each year, with a total of twenty four individuals affected over five separate outbreaks [90,91]. All of these outbreaks have been linked to the consumption of Spanish mackerel (*Scomberomorus commerson*) caught in New South Wales (NSW) coastal waters with Pacific ciguatoxin-1B concentrations of up to 1.0 mg·kg^−1^ (fish tissue) detected. Increasing ocean temperatures and an intensification of the East Australian Current have been hypothesised as potential reasons for the shift of this threat into more southern Australian waters [91]. 

## 6. Tetrodotoxins

Tetrodotoxin (TTX; Table 1) is found in a huge diversity of animals, including: fish, gastropods, crabs, marine flatworms, ribbon worms, arrow worms, annelid worms, starfish, sea slugs, octopus, newts and frogs. Puffer fish (“fugu”) is the most familiar, and most widely-studied, source of this toxin. In Australia, several marine animals are known to contain TTX, including the blue-ringed octopus (*Hapalochlaena lunulata*) [92] and “common” toadfish (*Tetractenos hamiltoni*) [92]. TTX has also been found in a scallop (*Patinopecten yessoensis*) cultivated in Japan [93], and recent work has shown trace amounts of TTX in pipis (*Paphies australis*) from New Zealand [53], oysters (*Crassostrea gigas*) from Greece [94] and various species of bivalve shellfish from the U.K. [95]. The presence of TTX in bivalve molluscan shellfish has very recently become an emerging phycotoxin issue in the EU. No regulatory limit has been established, although there is a current push for robust toxicological information and gathering of information relating to TTX levels found in shellfish. Careful monitoring of bivalve molluscs is required to evaluate the possibility of adverse effects on human health through inadvertent consumption of this potent neurotoxin.

Many derivatives of TTX have been isolated from animals [96]. These include epi-mers (4-epi-TTX, 6-epi-TTX), oxidized compounds (11-oxo-TTX, tetrodonic acid), deoxygenated compounds (5-deoxy-TTX, 11-deoxy-TTX, 4-epi-11-deoxy-TTX, 1-hydroxy-5,11-dideoxy-TTX, 5,6,11-trideoxy-TTX, 4-epi-5,6,11-trideoxy-TTX, 8-epi-5,6,11-trideoxy-TTX, 1-hydroxy-8-epi-5,6,11-trideoxy-TTX), compounds with the deletion of the methylene group at C-11 (11-nor-TTX-6(R)-ol, 11-nor-TTX-6(S)-ol, 11-nor-TTX-6,6-diol), anhydro derivatives (4,9-anhydro-TTX, 4,9-anhydro-6-epi-TTX, 4,9-anhydro-11-deoxy-TTX, 4,9-anhydro-8-epi-5,6,11-trideoxy-TTX, 1-hydroxy-4,4a-anhydro-8-epi-5,6,11-trideoxy-TTX), a compound modified at the C-11 hydroxymethyl group (chiriquitoxin) and a thiol conjugate (4-S-cysteinyl-TTX).

The origin of TTX in marine animals, whether from endogenous or exogenous sources, has been the subject of much debate and remains unresolved. There are reports that it can be derived from micro-algae, as it has been found in cultures of the dinoflagellate *Alexandrium tamarense* [97] and linked to a bloom of *Prorocentrum minimum* [94]. The accumulation of TTX in marine animals from the diet is plausible. However, levels in bacteria and marine sediments are low, and it remains difficult to account for the milligram quantities of TTX found in their tissues if derived from dietary sources.

## 7. Fish Killing Toxins

### 7.1. Karlotoxins

The biotoxins involved in blooms of the dinoflagellate *Karlodinium veneficum* (Syn: *Karlodinium micrum*, *Gymnodinium veneficum*) are called karlotoxins (KmTxs), with several described congeners [98]. KmTxs are lytic compounds that are highly active against blood cells, causing cell lysis (Table 1). These compounds appear to impact the gills of fish and other marine life. Hypoxia also may result from high density blooms. *Karlodinium veneficum* has been responsible for blooms linked to fish kills in southeast Australia, in particular a large-scale fish kill in Jervis Bay in January 2011, which resulted in the deaths of >10,000 fish, and rays in Hare Bay, in northern Jervis Bay (Shauna Murray, unpublished data). It was also linked to a fish kill in Lake Illawarra in 2000 [99].

In Western Australia, *K. veneficum* blooms regularly in the Swan River estuary (SRE) (1999, 2001, 2003, 2005, 2010 and 2012), often causing fish kills. A bloom (10,000 cells·mL^−1^) occurred in the SRE in March–July 2005, and high levels of KmTx were detected [54]. The bloom was localized over a bottom layer of hypoxic water in a stratified water column; elevated phosphate and ammonium were present, while nitrate levels were low, and salinity was 21–27 ppt. [54]. Blooms appear to develop under low flow conditions, and elevated flow rates appear to dissipate the blooms [54]. Whilst the distribution of *K. veneficum* in Australian waters is not completely known, it is likely to be widespread. Furthermore, blooms on the east and west coasts suggest a summer to autumn peak occurrence [6].

### 7.2. Amphidinolides and Other Fish Killing Toxins

*Amphidinium carterae* was the causative species for a very dense bloom (1.8 × 10^8^ cells·L^−1^), which occurred in the shallow, sandy, intermittently open coastal lagoon, on the northern beaches of Sydney in 2012 [100]. This bloom caused a visible water discoloration and co-occurred with the deaths of >300 individuals of *Acanthopagrus australis*, (*Mugil cephalus*) and *Anguilla reinhardtii*. Whilst many different types of toxic polyketide compounds (amphidinols, amphidinolides and others) are produced by strains of *Amphidinium carterae* and related *Amphidinium* species, including macrolides, short polyketides and long chain polyketides, a compound called luteophanol, chemically similar to amphidinol, was found to be produced by the strain of *Amphidinium carterae* during the Sydney bloom [100]. 

The opening of the lagoon to the ocean, as well as localized high nutrient levels preceded the observations of very high cell numbers. *A. carterae* is usually sediment-dwelling, but temporarily became abundant throughout the water column in this shallow (<2 m) sandy habitat. Histopathological results showed that the *Anguilla reinhardtii* individuals examined had damage to epithelial and gill epithelial cells. Fish kills due to this species have also been reported from Israel and Portugal, at similarly high cell abundance levels [98]. Although it is expected that this species is widespread in Australian coastal waters, there have been no studies on its distribution or seasonality to date [6].

## 8. Domoic Acid

Domoic acid (DA) is a toxic glutamate analogue produced by many species of *Pseudo-nitzschia*, which has worldwide distribution (Table 1). Several structural isomers of domoic acid (isodomoic acids A–H) and a stereoisomer (5′-epi-domoic acid) have been isolated from microalgae, diatoms, and/or shellfish. They cause the toxic syndrome known as amnesic shellfish poisoning (ASP), and a regulatory limit for DA of ≤20 mg·kg^−1^ is applied. DA is a water-soluble, polar, non-protein amino acid, which, due its rigid structure, binds to glutamate receptors in the central nervous system causing over excitation and, as a consequence, neuro-excitatory behaviour in humans, marine mammals, birds and fish ([101] and the references therein). Clinical symptoms range from gastrointestinal (nausea, vomiting, diarrhoea) to neurological (headaches, dizziness, disorientation, seizures, short term memory loss, permanent brain damage) and can be fatal.

Species belonging to the potentially toxic diatom genus *Pseudo-nitzschia* are a significant component of the phytoplankton community in Australian waters. Traditional light microscopy groupings indicate that the *P. seriata* group (cells >3 µm wide) is highest in abundance in the austral summer, autumn or spring (species dependent), while the *P. delicatissima* group (cells <3 µm wide) is in greatest abundance in winter and spring. Using morphological and molecular methods, seventeen species have been identified [7,102,103] with three species, *Pseudo-nitzschia australis*, *Pseudo-nitzschia cuspidata* and *Pseudo-nitzschia multistriata*, as confirmed DA producers (maximum DA 500 ng·mL^−1^, 11 pg DA per cell and 25.4 pg DA per cell, respectively) [7,56]. Interestingly *Pseudo-nitzschia multiseries*, a consistent producer of DA in all other strains tested throughout the world, proved to be the first nontoxic strain in Australia [7].

Not all species of *Pseudo-nitzschia* produce toxins, though, and species are extremely difficult to distinguish, with identification to the species level requiring the time-consuming identification of fine-scale characteristics only visible under an electron microscope. Moreover, elevated cell concentrations of *Pseudo-nitzschia* are commonly reported in the southeastern Australian oyster-growing estuaries [16]. In 2010, *Pseudo-nitzschia cuspidata* was responsible for the largest and longest toxic *Pseudo-nitzschia* blooms to date, closing the Sydney rock oyster (*Saccostrea glomerata*) harvest areas for 16 weeks (max. concentration of 34 mg·DA·kg^−1^ oyster tissue) and resulting in a significant financial loss to the AUDS$35 million industry (A. Zammit, personal communication, [7]). Being one of the most abundant and toxic *Pseudo-nitzschia* species, this *P. cuspidata* represents the greatest risk for aquaculture in Australian coastal waters. For this reason, a rapid molecular assessment tool for the real-time detection and quantification of *P. cuspidata* from environmental samples is required, so that suitable management can be implemented swiftly to reduce health risks and economic loss (see Knowledge Gaps and Conclusions). 

## 9. The Use of Molecular Genetic or ELISA Methods to Detect HABs

Over the past 10 years, many new technologies have been developed for the detection of the toxins involved in HAB events, as well as for the harmful algal species themselves. For the detection of harmful algal species, methods have centred on genetic techniques, such as qPCR directed at detecting species, or at genes involved in toxin biosynthesis (reviewed in [104]), HAB-species specific microarrays [105] and fluorescence in situ hybridisation methods [106]. In terms of chemical detection methods, many new fast techniques have been developed for PSTs, DSTs and ASTs, generally reliant on enzyme-linked immunosorbent assays (ELISA), including lateral flow devices and plate assays. Additionally, methods based on surface plasmon resonance (SPR) technology have been developed [107]. These have been the subject of several recent reviews [108,109]. In addition, new methods for the detection of these toxin groups have been developed to utilise LC-MS/MS equipment that is currently in use in shellfish monitoring programs [37,110].

In Australia, new technologies have been recently developed or researched for use in HAB monitoring, in response to the increased importance of HABs. Highly morphologically similar or identical species, which differ significantly in toxicity, can co-exist at several locations in Australia, [11], complicating the use of phytoplankton identification as an early warning system prior to shellfish contamination with toxins. In southern Australia, the dinoflagellates *A. australiense* (previously *A. tamarense* Group V), *A. pacificum* (*A. tamarense* Group IV) and *A. fundyense* (formerly *A. tamarense* Group I) can co-occur [12]. These three morphologically indistinguishable species often differ greatly in toxicity [14,17]. Methods that assay the presence of the gene sxtA4, which is a gene region found exclusively in PST-producing dinoflagellate species, have been developed and have shown promise as an early warning system for the detection of PSTs. This method has been used in NSW and Tasmania [111,112] and to assay oyster digestive glands directly as a fast screen for PSTs [113]. A comparison of multiple ELISA methods for the rapid detection of PST toxins has recently been conducted, aimed at improving the detection of PSTs in Tasmania, following the large-scale PST blooms that have occurred there (Dorantes-Aranda et al., 2017), and some of these rapid PST tests are now being used by shellfish farmers. In addition, several new methods have been developed for LCMS/MS instruments and are being used in Australian biotoxin laboratories, including new methods for PSTs [37] and for CTXs (Harwood et al., in prep).

## 10. Knowledge Gaps and Conclusions

The impacts of harmful algal blooms in Australian waters have clearly increased in recent years, as illnesses due to CFP have occurred in more southerly waters, and the first ever illnesses due to PSP have occurred in Tasmania. In addition, harvest area closures due to the detection of PSTs in shellfish have occurred for periods of up to four months at sites along the east coast of Tasmania, in 2012, 2015 and 2016, the longest harvesting area closures due to phycotoxins in Tasmania for at least 20 years. It appears that harmful algal blooms may have increased in frequency and duration in Australian coastal waters in recent years, although comprehensive datasets are only available from a few Australian states, in particular New South Wales. In this state alone, a total of 54 AST detections (max 20 in 2013), 27 DST detections (max 11 in 2013) and 102 PST detections (max 30 in 2010) from classified aquaculture harvest areas were recorded from 2004 to 2015. All of these detections, however, were below the regulatory limit across all toxin groups, with the exception of ASTs in *S. glomerata*, which exceeded the regulatory limit during one event (20 samples) in Wagonga Inlet in 2010 (NSW Food Authority, unpublished data, [104]). This highlights the need for improved data collection, in particular the collection and analysis of long-term datasets, so that trends can be assessed over the entire country. Nevertheless, research has intensified, into new detection technologies and monitoring strategies, and communication between the seafood industry, regulators and scientists has been enhanced through the creation of bodies, such as SafeFish (http://safefish.com.au/) and regular meetings, such as their Australian Shellfish Quality Assurance Advisory Committee (ASQAAC) Science Day (http://safefish.com.au/technical-program). 

The biotoxins of concern for Australia are ciguatoxins, paralytic shellfish toxins, okadaic acid and domoic acid, but palytoxins and karlotoxins are of growing importance. Whilst the majority of other phycotoxins (azaspiracids, yessotoxins, brevetoxin) and the cyclic imines (spirolides, gymnodimines, pinnatoxins and pteriatoxins) are routinely monitored, these toxins are not yet an issue for Australian coastal waters. The potential, however, for tetrodotoxin, maitotoxin and palytoxin contamination of seafood also needs to be investigated. Once toxin profiles are clarified and new techniques implemented and standardized, both requiring the need for purified standards, both intra- and inter-species toxicity comparisons are necessary. Future work also includes the validation and application of rapid, real-time molecular assays for the detection of harmful species and toxin genes. This, in conjunction with a better understanding of marine diversity and the life histories, physiological ecology, trophic interactions and environmental triggers of HAB species, may lead to better monitoring and management of these intensifying human and ecosystem health threats.

## Figures and Tables

**Table 1:** Emerging phycotoxin groups, chemical structure (example of the toxin within the group), causative microalgae, method of detection and reference. PSP, paralytic shellfish poisoning.

Phycotoxin Group	Chemical Structure (Example of Toxin within Group)	Microalgae	Method of Detection	Reference
Paralytic Shellfish Toxins	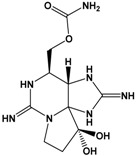 Saxitoxin (STX)	*Alexandrium fundyense* *Alexandrium pacificum* *Alexandrium australiense* *Alexandrium minutum* *Gymnodinium catenatum*	PSP Mouse bioassay	AOAC.OMA 959.08
			HPLC-FLD (pre-column oxidation)	AOAC-OMA 2005.06 [9]
			HPLC-FLD (post-column oxidation)	AOAC.OMA 2011.02 [10]
			Receptor Binding Assay	AOAC.OMA 2011.27 [36]
			LCMS	[37]
Diarrhetic Shellfish Toxins	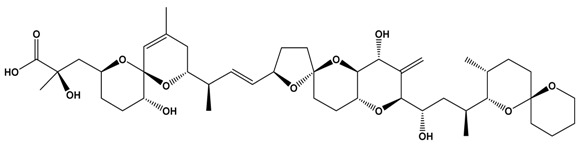 Okadaic acid (OA)	*Dinophysis acuminate* *Dinophysis tripos* *Dinophysis fortii* *Dinophysis caudate*	LC–MS (acidic mobile phase)	[38]
			LC-MS (alkaline mobile phase)	[39]
Pectenotoxins	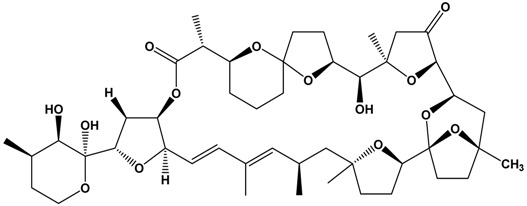 Pectenotoxin-2 (PTX-2)		HPLC/MS	[28,35]
			Protein Phosphatase Assay PP2A	[40]
Palytoxins, Ovatoxins and Ostreocins	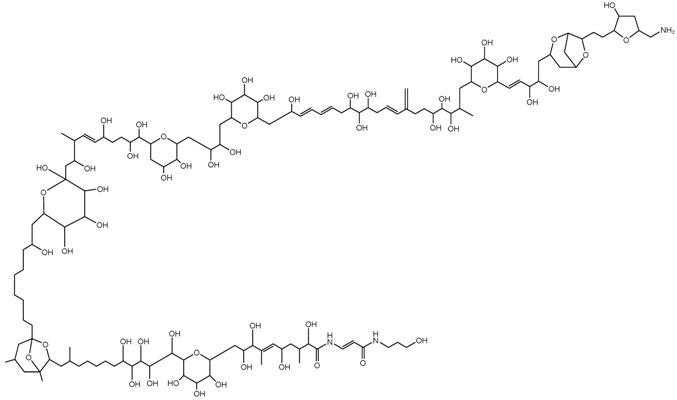 Palytoxin (PLTX)	*Ostreopsis* cf. *siamensis* *Ostreopsis ovata*	LC-MS (intact)	[41]
			LC-MS (substructures)	[42] [43]
Ciguatoxins	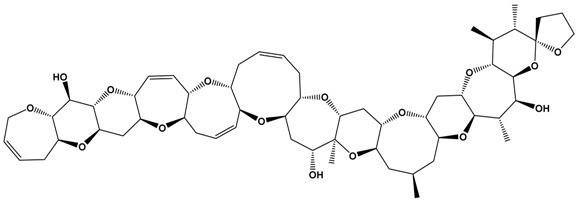 Ciguatoxin-3C (CTX-3C)	*Gambierdiscus* spp. *G. lapillus* sp. nov.	LCMS	[44,45,46,47]
			Receptor Binding Assay _R_ (radiolabelled)	[48]
Maitotoxins	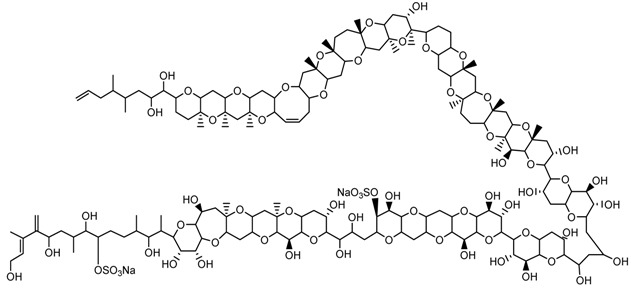 Maitotoxin-1 (MTX)		Receptor Binding Assay _F_ (fluorescent)	[49]
			Neuroblastoma Cell Assay (N2A)	[50]
Tetrodotoxins	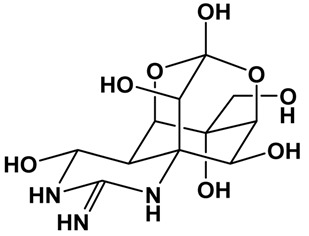 Tetrodotoxin (TTX)	*Alexandrium tamarense (?)* *Prorocentrum minimum (?)*	GC-MS	[51]
			LC-FLD	[52]
			LC-MS	[53]
Karlotoxins	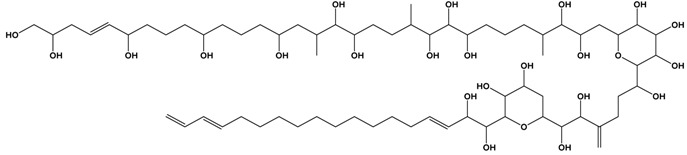 Karlotoxin-1 (KmTx-1)	*Karlodinium veneficum* (synonym: *Karlodinium micrum*, *Gymnodinium veneficum*)	LC-MS/MS	[54]
			HPLC-MS	[55]
Domoic Acid	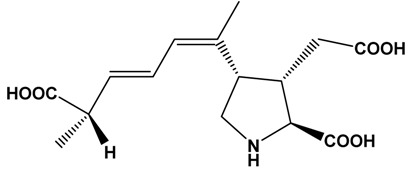 Domoic acid (DA)	*Pseudo-nitzschia australis* *Pseudo-nitzschia cuspidate* *Pseudo-nitzschia multistriata*	ELISA	AOAC-OMA 2006.02 [56]
			LC-MS/MS	[7]
			HPLC-UV	[57]

**Table 2:** Levels of diarrhetic shellfish poisoning (DSP) toxins (DSTs) in bivalve shellfish tested during a bloom of the toxic dinoflagellate *D. acuminata* in South Australia [35].

Sample (All Composites of 12 Individuals)	Toxin Concentration (mg/kg)				
	PTX-2	PTX-2 seco Acid ^a^	Okadaic Acid	Okadaic Acid Released by Hydrolysis	Total DSTs
Whole scallop	0.023	0.51	0.018	<0.050	0.041
Processed scallop ^b^	<0.010	0.037	0.014	<0.050	0.014
Whole razorfish	0.013	0.15	0.051	0.089	0.153
Processed razorfish ^c^	<0.010	0.023	<0.010	<0.050	<0.050
Whole oyster	0.11	0.79	0.023	0.12	0.253
FSANZ maximum limit for DSP toxins in bivalve molluscs					0.2

< denotes that the concentrations was less than the limit of detection and was considered to be nil. Pectinotoxin, PTX: FRANZ, Food Standards Australia New Zealand. ^a^ PTX-2 seco acid has been shown to be non-toxic to humans, so is not included in the total DSP calculation; ^b^ Processed scallops were tested with the viscera removed, with only adductor muscle and roe remaining; ^c^ Processed razorfish were tested with viscera removed, with only adductor muscle remaining.
